# Psychiatric morbidity among women in Norwegian prisons, 2010–2019: a register-based study

**DOI:** 10.1186/s12888-023-04886-7

**Published:** 2023-06-02

**Authors:** Vegard G Svendsen, Marianne Riksheim Stavseth, Torbjørn Skardhamar, Anne Bukten

**Affiliations:** 1 Norwegian Centre for Addiction Research, SERAF, University of Oslo, Building 45, Kirkeveien 166, Oslo, 0450 Norway; 2 Present Address: Division of Mental Health and Addiction, Oslo University Hospital, Oslo, Norway; 3 Department of Sociology and Human Geography, University of Oslo, Oslo, Norway

**Keywords:** Prison, Mental Health, Women, Psychiatric Morbidity, Substance Use Disorders, Dual Disorders, National Registry Data

## Abstract

**Purpose:**

Research suggests that women in prison have more mental health problems than men and are prone to suffer from more severe psychiatric disorders. This study utilizes national registry data to describe demographic and psychiatric gender differences in Norwegian prisons, and to investigate comorbid psychiatric disorders and time trends in psychiatric morbidity among women.

**Methods:**

Longitudinal data from the Norwegian Prison Release Study linked with the Norwegian Patient Registry and data from Statistics Norway provided information on health care utilization, socioeconomic status, and history of psychiatric disorders among all individuals (n_women_ = 5,429; n_men_ = 45,432) who were incarcerated in a Norwegian prison between 2010 and 2019.

**Results:**

Women were more likely than men to have a history of any psychiatric disorder (75% vs. 59%). Substance use disorders and dual disorders were highly prevalent in both genders, yet highest among women (56 and 38% respectively, versus 43 and 24% among men). From 2010 to 2019, we found a considerable increase in the 12-month prevalence of most diagnostic categories among women entering prison.

**Conclusion:**

Psychiatric and dual disorders are highly prevalent in Norwegian prisons, and especially among women. The proportion of women entering prison with a recent history of mental health problems has increased rapidly over the last decade. Women’s prison institutions need to adjust health and social services, and awareness about substance use and other psychiatric disorders in order to meet the increasing proportion of women facing these challenges.

**Supplementary Information:**

The online version contains supplementary material available at 10.1186/s12888-023-04886-7.

## Introduction

Women represent a minority in the prison context and are likely to present with needs and characteristics that are different from men’s [1, 2]. Research suggests that women in prison have more mental health problems than men and are prone to suffer from more severe psychiatric disorders [3–10]. They are at high risk of suicide and overdose death following release from prison [11, 12].

Studies that have investigated changes in psychiatric morbidity over time have found that the evident increase in mental health problems among people in prison over the last 10 to 20 years [13, 14] is notably more pronounced among women [15]. Of particular concern are the high rates of comorbid psychiatric conditions among women, especially the co-occurrence of substance use disorders (SUDs) with other psychiatric disorders – so called dual disorders [16–20]. People in prison with severe mental health problems, such as dual disorders, are more likely to also have other social, health, and behavioral problems, such as increased risk of self-harm [21], overdose and suicide death [22, 23], and recidivism [24, 25].

Given that prisons, in most instances, do not provide an ideal environment for treating or managing people with substance use disorders and/or mental illness [26], the accumulation of women with severe psychiatric conditions in prison represents a significant public health concern.

Furthermore, the lack of gender-specific knowledge about the occurrence, etiology, and unfolding of drug use disorders and mental health problems in the prison context might limit the provision of effective treatment and rehabilitation strategies [27, 28]. Indeed, acknowledgment of and knowledge about gender differences in incarcerated populations are central for developing and ensuring gender-informed policies and gender-specific interventions [7, 29]. To this end, it should also be noted that prison populations vary substantially between countries and across time, highlighting the importance of local, up-to date knowledge to sufficiently inform decision and policy makers.

As with other Scandinavian countries, there is little research or current empirical data on the mental health status and other important characteristics of women in the Norwegian prison system. Despite having one of the smallest prison populations and lowest incarceration rates in the world, Norway’s per-capita female prison population is one of the largest in Europe [30]. Still, little is known about who these women are and how their needs might be different from those of men in prison [31]. Meanwhile, voices from within the prison system have long sounded the alarm about the conditions in women’s prisons [32], and the high prevalence of severe mental health problems among its constituents [33–35].

With access to some of the most exhaustive and comprehensive national registries in the world, we propose a registry-based study to investigate the prevalence of psychiatric disorders in the Norwegian prison population. The first aim of this study is to describe key characteristics of the Norwegian prison population, and to examine whether there are gender differences in age, socio-economic background and the lifetime prevalence of psychiatric disorders. The second is to investigate, among women with a history of SUDs, which diagnoses tend to co-occur with SUDs and to what extent. The third aim is to explore time trends in sentencing and in the burden of psychiatric morbidity among women upon entry to prison.

## Methods

### Setting

Norway has a low imprisonment rate (59/100,000), which falls well below the European average (124/100,000) [30]. Women account for about 6.5% of the Norwegian total prison population (which is above the European median of 4.9%), at a gender specific imprisonment rate of 7.74/100,000 (vs. 109/100,000 for men). Norway has one of the most affluent social welfare systems in the world, with high levels of public health and other welfare services. The country’s correctional philosophy is motivated by rehabilitation and successful reintegration. In addition, as part of the effort to ensure high-quality health services for all, the right to universal health care, including drug treatment and rehabilitation, applies to all residents, regardless of incarceration status [36].

### Study population and observation period

We used data from The Norwegian Prison Release study (nPRIS [37]). The nPRIS cohort is drawn from the Norwegian Prison Registry [38, 39] and included all individuals serving time in a Norwegian prison between January 1, 2000 and December 31, 2019 (N = 129,902). Health records were available from 2009 onwards for persons 18 years and older.

### Selection and inclusion criteria

**Fig. 1:**
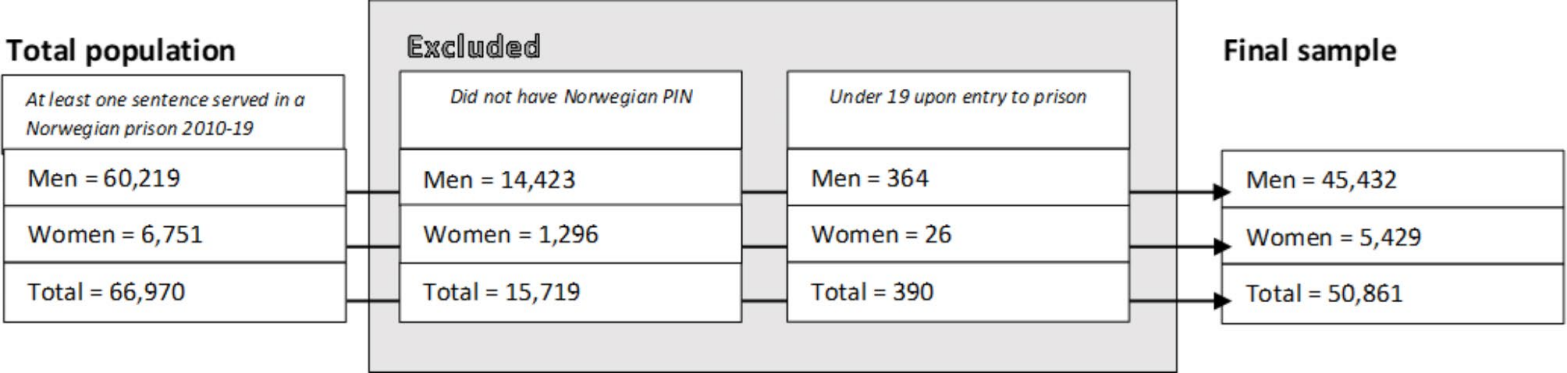
Flow chart and study population profile

Our cohort included women and men incarcerated in both high and low security units, in addition to pre-trial detention. People serving their sentence on probation (for example, community service or home detention) were not included in the nPRIS cohort. The nPRIS cohort was linked to other Norwegian national registries using a Norwegian personal identification number (PIN). Persons not holding a valid PIN were excluded from the study (Fig. 1).

We included individuals who upon entry to prison were aged 19 years or older and serving a prison sentence between January 1, 2010 and December 31, 2019. The date range allowed us to access health records one year prior to imprisonment. This resulted in a final sample including 5,429 women and 45,432 men (See Fig. 1.)

### Data sources

The study cohort was linked with the Norwegian Patient Registry (NPR) and socio-economic data from Statistics Norway (SSB) in the period January 1, 2009 to December 31, 2019. NPR is a national Registry of health care utilization which holds linkable information on all patients who have received secondary care for mental or somatic illnesses [40]. In addition to birth date, PIN and health care sector, NPR also includes primary and secondary diagnosis according to the *International statistical classification of diseases and related health problems, tenth revision (ICD-10)* [41], as well as date of admission and discharge. NPR contains information about persons aged 18 years or older, and has valid, linkable data beginning in 2009. SSB is the national statistical institute of Norway and responsible for collecting and producing statistics related to economy and demographics.

### Measures

This study employs several estimates of psychiatric morbidity, sentencing and socio-economic status (SES). These measures, and how they were operationalized, are detailed below. It should be noted that since any individual may have served more than one prison sentence during the observation period, measures will vary in whether they were used at the individual level (for which each person will have only one) or at the level of prison sentence (for which each person will have at least one, and possibly many).

#### Demography, SES and sentencing information

Age, gender and total number of prison sentences were collected at the individual level. Age was defined as age in years upon first entry to prison during the observation period. Number of prison sentences includes all imprisonments per individual during the observation period. Each sentence was categorized by sentence length. Short sentences were defined as below median sentence length, short to medium sentences were between the median and third quartile, and medium to long sentences were above the third quartile.

All SES variables were defined or aggregated at the individual level and operationalized as follows: Low education status was defined as not having completed any higher education (above year 10) at the latest recorded entry to prison during the observation period. Non-western background was defined as either having immigrated or being the child of two parents who emigrated from a country outside of EU/Schengen/UK, North America, New Zealand, or Australia. Status as parent of (an) underage child(ren) was operationalized as having at least one child under 18 years of age upon any entry to prison. Low-income background was defined according to the EU60 standard (i.e. as having an equivalized disposable household income below 60% of the median equivalized disposable household income in the general population [42]), in the preceding calendar year upon at least one entry into prison [43].

#### Psychiatric prevalence and dual disorders

We included all psychiatric diagnoses recorded in the NPR in this study, regardless of whether they were recorded as a primary or a secondary diagnosis. We then grouped them at the level of diagnostic categories with their corresponding ICD-10 codes: Alcohol and/or Substance Use Disorder ([F10] and/or [F11-19], excluding [F17]; Non-Affective Psychosis [F20-F23], [F25], [F28-29]); Bipolar Disorder ([F30-31]); Depressive and Mood Disorders ([F32-34], [F38-39]); Phobia and Anxiety Disorders ([F40-42], [F44]); Stress and Adjustment Disorders ([F43]); Somatoform and Other Related Disorders ([F45], [F48]); Eating Disorders ([F50]); Sleep Disorders ([F51]); Sexual Dysfunction Disorders ([F52]); Personality and Behavior Disorders ([F60-69], excluding [F67]); Hyperkinetic Disorders ([F90]). We defined dual disorders using previously established definitions from other studies and sorted them hierarchically at a general and specific level. Any dual disorder (general) was defined as having a history of SUD and at least one other psychiatric disorder from another diagnostic category [16]. Based on the criteria used by Baranyi et al. [17], we defined three specific sub-classes of dual disorders: comorbid SUD and Non-Affective Psychosis (as defined above); comorbid SUD and Major Depression ([F32-33]); and comorbid SUD and any Axis-I psychiatric disorder [F20-59] other than Non-Affective Psychosis or Major Depression.

#### Psychiatric morbidity

Psychiatric morbidity (defined as the history of at least one psychiatric or dual disorder) was assessed using two different approaches, with different temporal windows for detecting diagnoses registered in NPR. Both approaches relied on the same definitions and categorizations described above. To approximate a lifetime prevalence, we calculate a ten-year prevalence by including any psychiatric diagnosis in the NPR, regardless of when it was registered relative to the prison sentence. Dual disorders were also defined irrespective of time between diagnoses. In estimating the burden of psychiatric morbidity upon entry to prison, only diagnoses registered in the last 12 months prior to entering prison were included. For persons with more than one entry to prison during the observation period, a new 12-month prevalence was recorded for each new entry.

### Analysis

All analysis were performed in RStudio version 1.4.17. We conducted descriptive analysis separately for men and women. Among women, the history of recorded diagnoses, age and sentence length were further stratified by year. Categorical variables were summarized using percentage within each category and continuous variables (age and length of sentence) were summarized with median and interquartile range (IQR) due to skewed distributions. Gender differences were tested statistically using Wilcoxon rank sum test for continuous variables and Pearson’s Chi square test for categorical variables.

## Results

Between 2010 and 2019, 66,970 persons, of which 6,751 were women, served time in a Norwegian prison (Fig. 1). Of these, 15,719 (1,296 women) did not have a valid Norwegian PIN and could not be included in the study. A further 390 individuals (26 women) were under the age of 19 upon entry to prison and were therefore also excluded. The final study population consisted of n = 50,861 individuals (n = 5,429 women), accounting for 78,232 unique incarcerations (6,946 unique incarcerations for women).

### Background characteristics

Women (n = 5,429) were statistically significantly different from men (n = 45,432) in all measured characteristics, except educational background (Table 1). Slightly more women (44%) were parents to underage children compared to men (36%), and women were more likely to come from a low-income household (58% vs. 53%). Men served longer sentences than women and were more likely to have a history of several incarcerations during the observation period and to come from a non-western background.

Women were more likely than men to have a history of any psychiatric disorder (75 vs. 59%). Women also more prevalently had any SUD-diagnosis (56 vs. 43%), with the difference being largest for drug use disorders (DUDs) (48 vs. 34%) and less prominent for alcohol use disorder (AUD) (26 vs. 22%). More than one in three women (38%) had a dual disorder, while this was the case for one in four of the men (24%).

**Table 1:** Background characteristics and 10-year prevalence of any psychiatric disorder, SUD and any dual disorder in the entire sample (n = 50,861), stratified by gender

	Women n = 5,429	Men n = 45,432		*p-value*		
*Demographic variables*	n (%)	n (%)				
Median Age (IQR)	37 (28, 46)	34 (26, 45)		*< 0.001*		
Low Income Background	3,085 (58)	23,457 (53)		*< 0.001*		
Low education	1,706 (31)	14,250 (31)		*> 0.9*		
Non-Western Background	684 (13)	8,037 (18)		*< 0.001*		
Parent to underage child(ren)	2,365 (44)	16,517 (36)		*< 0.001*		
*Variables regarding incarcerations*						
History of more than one incarceration	1,019 (19)	12,909 (28)		*< 0.001*		
Median Sentence Length, days (IQR)	30 (20, 77)	49 (23, 121)		*<0.001*		
*History of psychiatric disorders* ^1^						
At least one psychiatric disorder	4,075 (75)	26,666 (59)		*< 0.001*		
Alcohol and Substance Use Disorder	3,031 (56)	19,394 (43)		*< 0.001*		
*Alcohol Use Disorder*	1,413 (26)	10,031 (22)		*< 0.001*		
*Drug Use Disorder*	2,614 (48)	15,576 (34)		*< 0.001*		
Any Dual Disorder	2,056 (38)	10,911 (24)		*< 0.001*		

^*1*^Over the observation period (10 years)

After SUDs, the most common psychiatric diagnostic categories among women were stress and adjustment disorders (29%), followed by depressive and mood disorders (27%), and phobia and anxiety disorders (23%) (Table 2). About one in six had a history of personality and behavior - or hyperkinetic disorders. Less prevalent diagnostic categories were eating disorders (2.8%) and somatoform and other disorders (2.2%). The large number of observations (n_sum_= 6,195) suggest that several individuals in the sample had a history of psychiatric comorbidity across diagnostic categories (Table 2).

**Table 2:** 10-year prevalence of the most common psychiatric diagnostic categories among women (n = 5,429) other than SUDs

Psychiatric Diagnostic Categories^1^	n (%)^2^
Stress and Adjustment Disorders	1,571 (29)
Depressive and Mood Disorders	1,466 (27)
Phobia and Anxiety Disorders	1,254 (23)
Personality and Behavior Disorders	936 (17)
Hyperkinetic Disorders	796 (15)
Non-Affective Psychosis	378 (7.0)
Bipolar Disorders	292 (5.4)
Eating Disorders	154 (2.8)
Somatoform and Other	117 (2.2)

^*1*^ Categories with less than 2% are not displayed; ^*2*^Categories are not mutually exclusive

### Dual disorders among women

As seen in Table 1, 38% of women had a history of dual disorders, and further details on those are provided in Table 3. Of those with dual disorders (n = 2,056), the most common second diagnosis were major depression disorder (46%), followed by any other Axis-I disorder (39%), and non-affective psychosis (16%).

Table 3 also shows diagnostic sub-categories of dual disorders involving Axis-I disorders other than major depression and non-affective psychosis. Of these 802 women, the most prevalent sub-categories were phobia and anxiety disorders (57%) and stress and adjustment disorders (57%) followed by bipolar disorders (11%), while the least prevalent were eating disorders (6%), somatoform and other disorders (4.5%), and mood and depressive disorders other than major depression (4%).

**Table 3:** Classes and prevalence (10 years) of dual disorders among women with any dual disorder (n = 2,056)

Classes of dual disorders^1^	n (%)
Comorbid SUD and Major Depression	940 (46)
Comorbid SUD and Non-Affective Psychosis	320 (16)
Comorbid SUD and Other Axis-I Disorder	802 (39)
*Phobia and Anxiety Disorders*	454 (22)
*Stress and Adjustment Disorders*	459 (22)
*Bipolar Disorders*	88 (4)
*Eating Disorders*	49 (2)
*Somatoform and Other*	36 (2)
*Other Depressive and Mood Disorders*	33 (2)

^*1*^Categories are not mutually exclusive

### Time trends in burden of psychiatric morbidity upon entry to prison

In narrowing the scope to include only diagnoses from the last 12 months prior to entry to prison, and comparing them by year, we observe that the absolute number of women with a recent history of psychiatric disorders has remained relatively stable between 2010 and 2019 (Table 4). However, given the significant decrease in new entries to prison over the same period, the prevalence over time shows a clear upward trend. By comparing our estimates from 2019 to 2010, we find a considerable increase in the prevalence of all diagnostic categories, except depressive and mood disorders. In terms of relative increase, we see that the greatest increase was for personality and behavior disorders (105% increase) and stress and adjustment disorders (100% increase), followed by alcohol use disorder (77% increase), phobia and anxiety disorders (66% increase) and drug use disorders (37% increase). The increase in dual disorders was also substantial (61%). An expanded version of Table 4, including all years and diagnostic categories, is available as an online supplementary file (S1).

**Table 4:** One-year prevalence of psychiatric morbidity by annual new entries to prison presented triannually, 2010–2019 (n = 6,946)

	2010 n = 848^*1*^	2013 n = 698^*1*^	2016 n = 771^*1*^	2019 n = 548^*1*^
History of at least one psychiatric diagnosis	339 (40%)	324 (46%)	417 (54%)	296 (54%)
Substance Use Disorders	267 (31%)	261 (37%)	341 (44%)	231 (42%)
Alcohol Use Disorder	53 (6.2%)	72 (10%)	89 (12%)	60 (11%)
Drug Use Disorders	233 (27%)	231 (33%)	301 (39%)	201 (37%)
Depressive and Mood Disorders	47 (5.5%)	48 (6.9%)	63 (8.2%)	30 (5.5%)
Phobia and Anxiety Disorders	35 (4.1%)	34 (4.9%)	45 (5.8%)	37 (6.8%)
Stress and Adjustment Disorders	51 (6.0%)	42 (6.0%)	70 (9.1%)	67 (12%)
Personality and Behaviour Disorders	33 (3.9%)	34 (4.9%)	65 (8.4%)	44 (8.0%)
Hyperkinetic Disorders	31 (3.7%)	29 (4.2%)	44 (5.7%)	35 (6.4%)
History of dual disorder	84 (9.9%)	86 (12%)	130 (17%)	88 (16%)

^*1*^n (%); Categories with average prevalence below 3% are not displayed

**Fig. 2:**
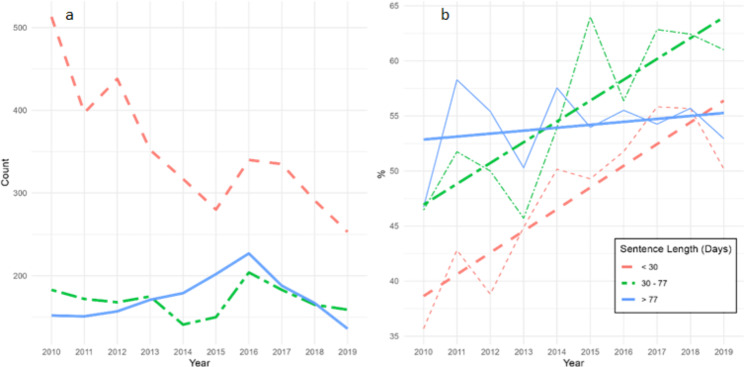
Time trends (2010–2019) by sentence length category in **(a)** annual new entries to prison and **(b)** 12-month prevalence of at least one psychiatric disorder, including a least squares best-fit line for each sentence length category

We stratified the annual number of new entries to prison and the 12-month prevalence of any psychiatric disorder by sentence length (Fig. 2) and found that the observed reduction in annual new entries to prison is largely driven by a substantial decrease in new entries to prison serving short sentences (from over 500 new entries in 2010 to 250 by 2019). In the same period, the number of new entries serving longer than short sentences has remained relatively stable (Fig. 2a). While the psychiatric morbidity among women serving medium to long sentences has remained high and relatively stable since 2010, the observed increase in psychiatric morbidity is most pronounced among those serving either a short (< 30 days) or short to medium (30–77 days) sentence (Fig. 2b).

## Discussion

Psychiatric disorders, SUDs and dual disorders are highly prevalent among persons who have served time in a Norwegian prison – and especially among women. Moreover, the proportion of women in prison with a recent history of mental health problems has increased considerably over the last decade. Indeed, since 2014 they have constituted the demographic majority among new entries to woman’s prison in Norway. The prevalence of any recently recorded psychiatric disorder in secondary care is almost ten times higher among women entering prison compared to women in the general population [44].

Our findings are generally consistent with the overall trends described in the literature. A substantial number of women, either at entry to prison [20] or during imprisonment [9], present with a current SUD or a dual disorder. We found that almost one-half of women entering prison in 2019 had a recent history of SUD, and that within the past 10 years 56% of the women in our sample had been diagnosed with SUD. Two in five women had a 10-year history of a dual disorder, among which the majority had a comorbid major depression, which represents one of the most common and severe variants of dual disorders [17, 45, 46].

The women in our cohort were more likely than men to have a history of any psychiatric disorder, including any SUD as well as any dual disorder. However, the utility in comparing crude prevalence estimates of men and women is greatly limited by the fact that we also observed significant gender differences in most other domains. This in turn speaks to the importance of recognizing the heterogeneity of prison populations, and that particularly women, on a group level, might be selected from a population that is systematically and importantly different from men.

The observed gendered disproportionality in psychiatric morbidity is in line with previous research, which has found that women in prison are more likely than men to report a lifetime history of mental health problems or to qualify for a current psychiatric diagnosis [3, 4] or a dual disorder [16]. The most notable difference in our sample compared with other reported findings is that the prevalence of alcohol use disorder was higher among women than among men – which is typically, although marginally, the other way around [47].

A longitudinal cohort study by Chang and colleagues is the only other study in the current literature sufficiently comparable to our methodology and geography [10]. Their study uses national registry data to investigate the association between psychiatric disorders and mortality after release from Swedish prison. They describe the history of psychiatric disorders, stratified by gender, as well as a selection of socio-economic factors. In general, our findings are consistent with those reported by Chang et al. [10], which also found higher levels of psychiatric disorders in women relative to men, including alcohol use disorder. What our study adds to the Scandinavian literature is the inclusion of dual disorders and observed time trends in sentencing and psychiatric morbidity.

The increase within in the prison context of mental health problems such as SUDs [47] as well as psychotic [14] and dual disorders [13, 17] has been observed by other researchers, but remains not very well understood. The underlying mechanisms likely to be driving this phenomenon are both highly complex and context dependent. To our knowledge, a recent Australian study by Browne et al. is the only other study that has observed and reported on this increase in terms of gender differences [15]. That study, similar to ours, found a clear increase in the prevalence of psychiatric disorders in prison, particularly among women. However, while their findings could partly be accounted for by a general increase in mental health problems within the general population of Australia, no comparable trend has been observed in Norway [44].

We propose that the demographic changes detected in our data might reflect recent developments in Norwegian sentencing practice, especially the increased use of electronic monitoring (EM) as an alternative to imprisonment [48]. In Norway, serving an entire sentence on EM is reserved for shorter prison sentences, which is likely the primary reason behind the notable decrease in the number of shorter prison sentences observed in our own data.

### Strengths and limitations

The novelty and main strength of this study lies in the unique quality of the available data material [49]. With access to reliable, high quality registry data, spanning a decade worth of consistent observations on a complete prison population, we have been able to present some of the most extensive psychiatric epidemiological findings in the field of medical prison research to date.

Nevertheless, the use of registry data, especially for estimating disease prevalence, also introduces several limitations to this study. First, it might be more appropriate to consider our estimates to reflect a person’s history of secondary health care utilization rather than a prevalence per se - and that these estimates are likely underestimates of the true prevalence. Moreover, the trends and estimates presented in this study might be sensitive to several unmeasured factors, such as temporal changes and potential gender differences in health seeking behavior and the availability of health services, which might have influenced the probability of having a diagnosis recorded in NPR.

Second, all persons with a residential permit in Norway have a PIN, which is a prerequisite for linking national registry data. However, the prison population also consists of a large minority of persons without residential permits who were therefore excluded from this study (24% of the initial sample). As such, our final sample is only partially representative of the Norwegian prison population. It is likely that the inclusion of non-residents, had it been possible, would have influenced our results. Third, as we did not have access to information about onset of any disorder our study precludes assumptions about causal associations.

### Clinical implications and conclusions

We observed that despite a substantial reduction in the number of new entries to prison between 2010 and 2019—especially among the shortest prison sentences—the number of women entering prison with a recent psychiatric diagnosis has remained relatively stable. Consequently, the proportion of women entering prison with a recent history of at least one psychiatric disorder has increased substantially.

We encourage law and policy makers as well as providers of correctional services and health care to be particularly attentive to the potential implications of this scenario. A declining prison population notwithstanding, women with the most severe cases of substance use and/or other mental health problems—who in turn are likely to require the most treatment and support—are still ending up in prison. As such, correctional institutions might need to scale up their psychiatric and psychological treatment capacity and increase staff awareness and competence in mental health and dual disorders in the prison context. In addition, we suggest improved screening procedures, such as the application of validated instruments to detect mental health and/or substance use issues at entry to prison [50, 51]. Finally, we encourage enhanced co-operation between health and correctional services as necessary measures to facilitate appropriate levels of health care delivery - both during and after imprisonment.

Prison can never provide the ideal environment for institutionalizing persons with severe psychiatric disorders. Nevertheless, a prison sentence represents a unique window of opportunity for some of society’s most marginalized individuals to encounter health care services and treatment options [52]. At a minimum, our prison institutions should be optimized for both identifying and caring for those who are incarcerated with psychiatric disorders.

## Electronic supplementary material

Below is the link to the electronic supplementary material.

Supplementary Material 1

## Data Availability

This population study used individual-level data from The Norwegian Prison Registry (held by the Directorate of Norwegian Correctional Service), the Norwegian Patient Registry (held by the Norwegian Institute of Public Health) and various socio-economic registry data held by Statistics Norway. The ethical approval of this research project does not include permission to share the raw data publicly. Qualifying researchers can apply for access to relevant data from the Norwegian Institute of Public Health, Statistics Norway and the Directorate of Norwegian Correctional Service on approval from the Regional Committees for Medical and Health Research Ethics.
