# Supplementary material for: Psychiatric morbidity among women in Norwegian prisons, 2010–2019: a register-based study
Source: BMC Psychiatry. 2023 Jun 2;23:390. doi: 10.1186/s12888-023-04886-7 (PMC10236692; doi:10.1186/s12888-023-04886-7)
Supplement: Supplementary file 1 — Supplementary Material 1 [file 12888_2023_4886_MOESM1_ESM.pdf]

Supplementary table 1 (S1), One-year prevalence of psychiatric morbidity by annual new entries to prison presented triannually, 2010-2019 (n= 6,946).

**Characteristic**	**2010**, n = 848[1]	**2011**, n = 720[1]	**2012**, n = 763[1]	**2013**, n = 698[1]	**2014**, n = 637[1]	**2015**, n = 632[1]	**2016**, n = 771[1]	**2017**, n = 706[1]	**2018**, n = 623[1]	**2019**, n = 548[1]
Age, years	35 (26, 44)	37 (27, 45)	36 (27, 44)	37 (28, 46)	37 (28, 45)	37 (29, 46)	38 (30, 47)	39 (30, 48)	38 (30, 47)	39 (29, 47)
History of at least one psychatric diagnosis	339 (40%)	347 (48%)	341 (45%)	324 (46%)	338 (53%)	343 (54%)	417 (54%)	404 (57%)	358 (57%)	296 (54%)
Substance Use Disorders	267 (31%)	269 (37%)	271 (36%)	261 (37%)	284 (45%)	280 (44%)	341 (44%)	308 (44%)	286 (46%)	231 (42%)
Alcohol Use Disorder	53 (6.2%)	59 (8.2%)	60 (7.9%)	72 (10%)	57 (8.9%)	78 (12%)	89 (12%)	89 (13%)	71 (11%)	60 (11%)
Drug Use Disorders	233 (27%)	241 (33%)	248 (33%)	231 (33%)	252 (40%)	245 (39%)	301 (39%)	271 (38%)	257 (41%)	201 (37%)
Depressive and Mood Disorders	47 (5.5%)	48 (6.7%)	56 (7.3%)	48 (6.9%)	42 (6.6%)	30 (4.7%)	63 (8.2%)	52 (7.4%)	36 (5.8%)	30 (5.5%)
Phobia and Anxiety Disorders	35 (4.1%)	37 (5.1%)	33 (4.3%)	34 (4.9%)	32 (5.0%)	41 (6.5%)	45 (5.8%)	53 (7.5%)	43 (6.9%)	37 (6.8%)
Stress and Adjustment Disorders	51 (6.0%)	42 (5.8%)	37 (4.8%)	42 (6.0%)	43 (6.8%)	54 (8.5%)	70 (9.1%)	82 (12%)	71 (11%)	67 (12%)
Hyperkinetic Disorders	31 (3.7%)	34 (4.7%)	41 (5.4%)	29 (4.2%)	26 (4.1%)	33 (5.2%)	44 (5.7%)	37 (5.2%)	46 (7.4%)	35 (6.4%)
Personality and Behaviour Disorders	33 (3.9%)	41 (5.7%)	38 (5.0%)	34 (4.9%)	44 (6.9%)	42 (6.6%)	65 (8.4%)	71 (10%)	60 (9.6%)	44 (8.0%)
Non Affective Psychosis	10 (1.2%)	13 (1.8%)	16 (2.1%)	14 (2.0%)	14 (2.2%)	15 (2.4%)	30 (3.9%)	17 (2.4%)	23 (3.7%)	14 (2.6%)
Bipolar Disorders	20 (2.4%)	17 (2.4%)	13 (1.7%)	8 (1.1%)	11 (1.7%)	11 (1.7%)	16 (2.1%)	16 (2.3%)	11 (1.8%)	14 (2.6%)
Comorbid SUD with Major Depression	19 (2.2%)	12 (1.7%)	21 (2.8%)	17 (2.4%)	15 (2.4%)	12 (1.9%)	20 (2.6%)	18 (2.5%)	18 (2.9%)	12 (2.2%)
Comorbid SUD with Non-Affective Psychosis	6 (0.7%)	10 (1.4%)	12 (1.6%)	12 (1.7%)	11 (1.7%)	11 (1.7%)	20 (2.6%)	10 (1.4%)	18 (2.9%)	12 (2.2%)
Comorbid SUD with other AXIS-I disorder	49 (5.8%)	40 (5.6%)	41 (5.4%)	47 (6.7%)	34 (5.3%)	57 (9.0%)	69 (8.9%)	68 (9.6%)	55 (8.8%)	60 (11%)
History of dual disorder	84 (9.9%)	82 (11%)	83 (11%)	86 (12%)	82 (13%)	100 (16%)	130 (17%)	120 (17%)	106 (17%)	88 (16%)

[1]Median (IQR); n (%)
